# Genes of Experience: Explaining the Heritability of Putative Environmental Variables Through Their Association with Behavioural and Emotional Traits

**DOI:** 10.1007/s10519-013-9591-0

**Published:** 2013-03-22

**Authors:** Tom A. McAdams, Alice M. Gregory, Thalia C. Eley

**Affiliations:** 1 MRC Social Genetic and Developmental Psychiatry Centre, Institute of Psychiatry, King’s College London, P.O. Box 80, De Crespigny Park, Denmark Hill, London, SE5 8AF UK; 2 Department of Psychology, Goldsmiths University of London, London, UK

**Keywords:** Aggression, Anxiety, Delinquency, Depression, Gene–environment correlation, Maternal negativity, Negative life events, Oppositionality, Paternal negativity

## Abstract

An increasing body of evidence shows that many ‘environmental’ measures are heritable, indicating genetic involvement in environmental exposure (or *gene*–*environment correlation*). In the present study we attempt to clarify why three such ‘environmental’ measures (maternal negativity, paternal negativity and negative life events) are consistently found to be heritable. Through multivariate genetic analysis of a sample of adolescent twins from the UK we show that the heritability of these putative environmental measures can be explained via their association with five behavioural phenotypes: oppositionality, delinquency, physical aggression, depression and anxiety. This is consistent with the notion that being genetically susceptible to certain behavioural difficulties could lead to exposure to certain life events, and this may account for the reported heritability of ‘environmental’ measures. Results are discussed in the context of possible active, evocative and passive gene–environment correlations.

## Introduction

Genes play an important role in explaining the appearance, behaviour and personality characteristics of people (Plomin et al. 2008). Behavioural geneticists and evolutionary biologists have long-since noted that genes also operate ‘beyond the skin’ and can play an important role in shaping the environment that an individual experiences (Dawkins 1982; Kendler and Baker 2007). Evidence for such phenomena in humans comes primarily from twin and family studies (Jaffee and Price 2007; Kendler and Baker 2007). Such studies have demonstrated the heritability of numerous environmental measures including the home environment (Saudino and Plomin 1997), life events (Button et al. 2008), parental discipline (Button et al. 2008), and bullying victimisation (Ball et al. 2008). Molecular genetic studies have also linked candidate genes with marital status (Dick et al. 2006), popularity within the peer group (Burt 2008), and negative parenting experiences (Lucht et al. 2006). Such findings are indicative of gene–environment correlation (rGE)—a relationship between genotype and environment.

### The relationship between behaviour and environment

Twin studies often suggest that controllable life events are more heritable than uncontrollable life events (Kendler and Baker 2007). This implies that genetic factors have greatest impact upon environmental measures that are influenced by the behaviour of the individual. In other words, it seems likely that genes operate on the environment by first affecting behaviour that in turn influences the environment.

One way of assessing the possible role of behaviour in explaining the heritability of environmental measures is to examine the degree of genetic overlap between environmental measures and behavioural phenotypes (Saudino and Plomin 1997; Pike et al. 1996). Taking this approach researchers have detected genetic overlap between (amongst others) parental negativity and depression (Pike et al. 1996), peer deviance and conduct problems (Button et al. 2007), and life events and personality (Saudino et al. 1997). Overlap of this sort suggests that the heritability of environmental measures may be explicable through their association with heritable behavioural phenotypes. That is, if the heritability of negative life events is partially accounted for by its association with delinquent behaviour, this would be consistent with the notion that the genetically influenced delinquency of the adolescent leads to (or is associated with) an environment in which negative life events are likely to occur.

Most twin studies assessing genetic overlap between environmental measures and behavioural phenotypes have used bivariate models to partially account for the heritability of environmental measures (e.g. Pike et al. 1996). However, in some cases researchers have included several behavioural phenotypes and in doing so have explained a large portion of the heritability of the environment. For example, Saudino and Plomin (1997) showed that the heritability of the home environment could be entirely accounted for by its association with the child’s temperament and cognitive ability. That is, genetic factors involved in temperament and ability overlapped with those associated with the home environment to the extent that no residual genetic variance remained once this overlap was accounted for. However, neither phenotype alone was enough. Such a multivariate approach holds great promise: we may be able to entirely explain the heritability of environmental measures via their association with multiple heritable traits. It is important that such attempts are made to explain ‘why’ studies find environmental variables to be heritable—by highlighting likely pathways and mechanisms.

Although bivariate genetic analyses may indicate that (for example) elements of the family environment are genetically correlated with both antisocial behaviour and depression (Pike et al. 1996), we cannot assume that including both of these behavioural phenotypes in the same analysis will explain more of the heritability of parental negativity than does either one alone. This is because antisocial behaviour and depression are also genetically correlated (Rowe et al. 2008). This means that their contributions to the heritability of parental negativity are not independent of one another. In other words, their joint contribution may be less than the sum of their individual contributions. As such, studies are needed that incorporate multiple behavioural phenotypes into multivariate genetic analyses, allowing researchers to assess the extent to which behavioural measures together account for the heritability of environmental measures.

### The current study

In the current study we examine three environmental measures previously reported as heritable–maternal negativity (negative behaviours and punitive discipline directed towards the child), paternal negativity and negative life events.

We focus on these putative environmental measures because they are frequently reported to be heritable (Kendler and Baker 2007; Button et al. 2008), they are prevalent, and they are often associated with psychopathology in child and adolescent samples. For example, negative life events have been associated with antisocial behaviour (Wiesner and Windle 2004), depression (Kendler et al. 1999; Patton et al. 2003), and the overlap between them (Kim et al. 2003; Rowe et al. 2006). Parental negativity has been linked to anxiety disorders (Hudson and Rapee, 2001) and low self control in children (Cecil et al. 2012). As well as being phenotypically related to psychopathology, negative life events and parental negativity share genetic variance with commonly occurring behavioural and emotional problems: Genetic overlap has been reported between negative life events and depression (Kendler and Karkowski-Shuman 1997), anxiety (Boer et al. 2002), and antisocial behaviour (Button et al. 2008). Twin studies have also shown parental negativity to be genetically correlated with depression (Pike et al. 1996), and antisocial behaviour (Button et al. 2008; Narusyte et al. 2011; Pike et al. 1996).

In the present study we therefore investigate the hypothesis that maternal negativity, paternal negativity and negative life events are heritable at least in part because of their association with several behavioural phenotypes they are often associated with; depression, anxiety, and antisocial behaviour. Because recent research demonstrates that antisocial behaviour is heterogeneous (Burt and Neiderhiser 2009; Rowe et al. 2006, 2008; Tremblay 2010), and subtypes may differ in their heritability (Burt 2009), correlates (Burt and Donnellan 2008) and developmental patterns (Burt and Neiderhiser 2009; Tremblay 2010), we distinguish between three distinct forms of antisocial behaviour—physical aggression, non-violent delinquency, and oppositionality.

We use a sample of adolescent twins in our study. By using an adolescent twin sample we are able to assess the role of adolescent’s genes in their experiences of parental negativity and life events. During adolescence the environment exerts considerable influence on development at the same time as the individual begins to exert influence on their environment. As such adolescence is an ideal developmental period in which to study genetic involvement in environmental exposure. To our knowledge this is the first attempt to entirely account for the heritability of putative environmental measures via their association with multiple behavioural phenotypes.

## Method

### Sample

The present sample consists of adolescent twins who participated in wave 2 of the G1219 study—a longitudinal study of twins and their families. Full details of the study are given elsewhere (McAdams et al. 2013). At the first wave of the study participants were 3,640 adolescents aged 12–19. At the second wave 2,647 individuals took part (73 % of the original sample). Wave 2 questionnaires were completed an average of 8 months (range = 0.8–22 months) after initial contact. Wave 2 was used for the present analyses as it is the wave containing the most environmental measures.

Analyses included 150 male MZ twin pairs, 178 female MZ twin pairs, 133 male DZ twin pairs, 178 female DZ twin pairs, and 463 opposite-sex DZ pairs. Sixty-nine percent of the sample was female, the mean age of the sample was 14.58 years old (range 13–17, SD = 1.36).

Data were collected via postal questionnaire. The first wave of data was weighted (at the family level) to match the distribution of educational qualifications observed in a nationally representative sample (Meltzer et al. 2000; for full details of the first wave weighting see Rowe et al. 2006). The second wave was weighted (at the family level) according to predictors of attrition, using the inverse of the predicted probability of families remaining in the study at wave 2. Predictors were parental education, housing tenure, and child sex (girls being most likely to respond). This response weight was multiplied by the wave 1 sampling weight to provide a single weighting variable. In the present study substantively identical results were obtained when analyses were run with and without this weight.

All participants aged ≥16 provided informed consent. For those <16 years old informed consent was obtained from parents. Ethical approval for the study was provided by the Research Ethics Committee of the Institute of Psychiatry and the South London and Maudsley NHS trust.

### Measures

All behavioural phenotypes and environmental measures were assessed via twin self-report questionnaires.

#### Antisocial behaviour

Physical aggression, oppositionality and non-violent delinquency were measured using items from the Youth Self-Report questionnaire (Achenbach 1991). Scales comprised 3, 8, and 11 items respectively. Examples include “I physically attack people”, “I argue a lot”, and “I lie or cheat”. Response options were ‘not true’ (0), ‘somewhat true’ (1), or ‘very true’ (2), regarding behaviour during the last 6 months. Internal reliability was acceptable: Cronbach’s alpha was 0.65 for physical aggression; 0.76 for oppositionality; and 0.70 for delinquency.

#### Depressed mood

Depressed mood was measured using the Moods and Feelings Questionnaire—short version (Angold et al. 1995). Participants responded to 13 self-report items assessing how often they have experienced signs of depression over the previous 2 weeks. Examples include “I felt miserable or unhappy”. Response options were never (0), sometimes (1), often (2), always (3). Cronbach’s alpha was 0.89.

#### Trait anxiety

Trait anxiety was measured using the Spence Children’s Anxiety Scale (Spence 1998), comprising 38 self-report items assessing the frequency with which participants experience feelings of separation anxiety, social phobia, obsessive compulsive behaviours, panic, fear of physical injury and generalised anxiety. Examples include “I worry what other people think of me”. Response options were never (0), sometimes (1), often (2), always (3). Cronbach’s alpha was 0.91.

#### Parental negativity

Parental negativity was assessed using the Negative Sanctions subscale adapted from a previously well-validated parent–child relationship measure (Dunn et al. 2003; Hetherington and Clingempeel 1992). Five items assessed children’s perceptions on how common it was for their parents to yell at them, take away their privileges, make fun of them, and act authoritatively towards them (2 items). This was repeated for each parent, resulting in paternal and maternal negativity scales. Example items include “How common is it for your mum to yell at you about something you did wrong?” Response options were very uncommon (0), uncommon (1), somewhat common (2), common (3), very common (4). Cronbach’s alpha was 0.73 for paternal negativity and 0.67 for maternal negativity.

#### Negative life events

Negative life events were measured using items from the life events for adolescents scale (Coddington 1984). Twelve items assessed whether or not a series of negative dependent-life-events had happened to respondents in the previous year (see Rowe et al. 2006). Examples include “suspension from school” and “being sent away from home”. Cronbach’s alpha for the scale was 0.59 (range 0–9).

### Analyses

#### Phenotypic analyses

Phenotypic relationships between variables were explored using the survey models of Stata 10 (StataCorp 2007). Parameter estimates, standard errors and *p* values were adjusted for sampling weight and the non-independence of observations from the same family (treating the family as the primary sampling unit). Resultant models allow for intragroup correlation and relax the requirement that observations be independent of one another. They are therefore suitable for the analysis of samples of related individuals.

#### Genetic analyses

Genetic analyses were conducted using the structural equation modelling programme OpenMx (Boker et al. 2011). The twin method involves comparing intra-familial similarity in MZ and DZ twin pairs. MZ twins share all genetic effects, whereas DZ twins share on average 50 % of their segregating genes. Analyses involve decomposing variance/covariance into influences due to additive genetic (A), shared environment (C environmental factors that make members of a twin pair alike) and non-shared environment factors (E environmental factors that make members of a twin pair different to one another).

**Fig. 1:**
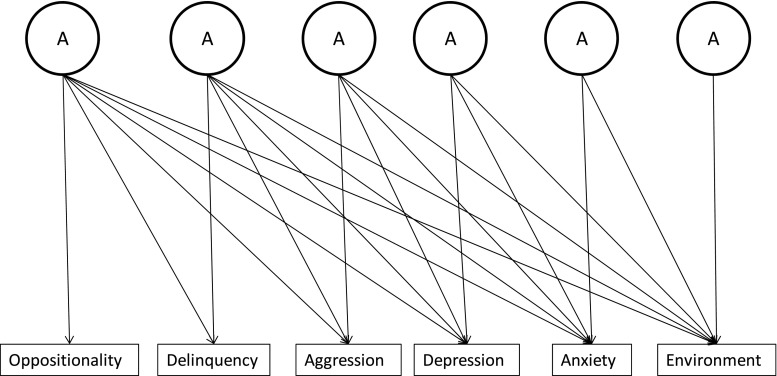
Cholesky decomposition model. Going from *left* to *right* the first A factor accounts for genetic variance common to all variables, the second A factor accounts for any remaining variance in the latter 5 variables not accounted for by the first factor, and so on. The final factor accounts for residual variance in the final (*extreme right*) variable not shared with any other variable in the model

Three Cholesky decomposition models (one for each environmental measure) were employed to assess whether the heritability of each of our environmental measures was accounted for via their association with the behavioural phenotypes (see Fig. 1). Environmental measures were entered as the final variable in the model (the variable to the far right in Fig. 1). This meant that variance in the environmental measure that is shared with each of the behavioural phenotypes would be accounted for. As such the final A (or C or E) factor in the model would comprise variance unique to the environmental measure. If the path estimate for this factor was greater than zero then this would indicate that our behavioural phenotypes had not accounted for all of the variance in our environmental measure. We were also interested in the nature of bivariate associations within the multivariate model (e.g. whether life events had a stronger genetic correlation with depression or delinquency). We therefore transformed the Cholesky decompositions into the more easily interpretable correlated factors solution (Loehlin 1996) and report bivariate genetic correlations and factor loadings. We report estimates of residual genetic variance from the Cholesky decompositions in the text.

Models were fitted using −2log likelihood simultaneously in 5 groups; MZ male, DZ male, MZ female, DZ female, and DZ mixed-sex pairs. Nested models were tested using the *χ* ^2^ fit statistic to test for significant change in model fit. Akaike’s Information Criterion (AIC) was also used in tandem with the −2LL. Broadly speaking, when comparing models a smaller AIC value suggests a better (or more parsimonious) fit. The AIC can be particularly useful when comparing models that are not nested (e.g. ACE models vs. saturated models). Prior to genetic analyses square-root transformations were applied to all variables to normalise their distributions. Residuals were taken to account for the effects of sex and age on all variables.

## Results

Means and standard deviations, and the results of regression analyses examining the effects of age and sex on each of the study variables, are presented in Table 1. Maternal and paternal negativity were both negatively related to age indicating that in our sample younger adolescents reported experiencing more negativity than did older adolescents. The experience of negative life events was unrelated to age. None of the environmental phenotypes differed between girls and boys. For the behavioural phenotypes delinquency was positively related to age, whereas anxiety, oppositionality and physical aggression were negatively related to age. Depression was unrelated to age. Girls reported experiencing more depression and anxiety symptoms than boys. Boys reported higher levels of delinquency, and physical aggression than girls. Oppositionality did not differ between the sexes.

**Table 1:** Descriptive statistics and tests for sex and age effects on all variables included in the study

	Boys		Girls		Sex	Age
	M	SD	M	SD	β (95 % CI)	β (95 % CI)
Maternal negativity	7.29	3.84	7.72	3.90	0.06 (−0.01, 0.13)	−0.10 (−0.17, −0.04)*
Paternal negativity	7.01	4.50	7.02	4.43	0.01 (−0.06, 0.07)	−0.12 (−0.19, −0.05)*
Negative life events	1.15	1.37	1.22	1.41	0.02 (−0.04, 0.09)	0.03 (−0.04, 0.09)
Depression	6.70	5.55	8.58	7.29	0.14 (0.07, 0.21)*	0.03 (−0.04, 0.10)
Total anxiety	20.78	12.69	25.97	14.21	0.19 (0.13, 0.25)*	−0.07 (−0.14, −0.01)*
Oppositionality	3.94	2.96	4.05	2.98	0.02 (−0.04, 0.09)	−0.08 (−0.15, −0.01)*
Delinquency	3.29	2.90	2.65	2.43	−0.25 (−0.36, −0.13)*	0.47 (0.35, 0.59)*
Physical aggression	0.83	1.24	0.44	0.87	−0.16 (−0.22, −0.10)*	−0.08 (−0.15, −0.01)*

*Sex*: boys = 1, girls = 2

* Significant to at least *p* < 0.05

Pair-wise correlations between phenotypes are presented in Table 2. Correlations were generally weak-to-moderate. Of the environmental measures negative life events tended to have the strongest correlations with the behavioural phenotypes, with correlations ranging from 0.17 (anxiety) to 0.48 (delinquency). Correlations between maternal negativity and the behavioural phenotypes ranged from 0.18 (physical aggression) to 0.38 (oppositionality). Of the three environmental measures paternal negativity had the weakest relationships with the behavioural phenotypes, with correlations ranging from 0.12 (delinquency) to 0.23 (oppositionality). Sex differences in the magnitude of correlations were not significant (95 % confidence intervals were overlapping).

**Table 2:** Pair-wise correlations between environmental measures and behavioural phenotypes

	M.Neg.	P.Neg.	N.L.E.	Dep.	Anxiety	Oppo.	Delinq.
Paternal negativity	0.53						
Negative life events	0.26	0.17					
Depression	0.27	0.18	0.32				
Anxiety	0.23	0.17	0.17	0.59			
Oppositionality	0.38	0.23	0.36	0.45	0.24		
Delinquency	0.24	0.12	0.48	0.36	0.12	0.62	
Physical aggression	0.18	0.14	0.29	0.22	0.03 (ns)	0.56	0.59

All correlations significant to at least *p* < 0.01, except for *ns* not significant

*M.Neg.* maternal negativity, *P.Neg.* paternal negativity, *N.L.E.* negative life events, *Dep.* depression, *Oppo.* oppositionality, *Delinq.* delinquency

### Genetic analyses

Univariate genetic analyses for all of the variables included in the current study have been presented elsewhere (see Button et al. 2008; Rowe et al. 2008; Zavos et al. 2010). Briefly, each of the environmental variables is ~40 % heritable. The majority of remaining variance is attributable to environmental influences not shared by family members, with some evidence of shared environmental influences. Sex differences for all variables are minimal and/or largely non-significant.

### Multivariate genetic analyses: accounting for the heritability of environmental measures

Three separate Cholesky decomposition models were run, one for each environmental variable. Full details of the model fitting process, including saturated model fit is included in the Appendix (Tables 3, 4, 5). Cholesky decompositions were transformed into correlated factors solutions for ease of interpretation.

**Table 3:** Model fitting results for the maternal negativity Cholesky decomposition

Model	−2LL (DF)	AIC	*χ* ^2^ (df)	*p*
1. Saturated	26496.63 (10485)	5526.63		
2. Cholesky with scalars on all variables	27058.22 (10854)	5350.22		
**2a. Cholesky with scalars on depression and physical aggression**	**27062.47 (10858)**	**5346.47**	**4.24 (4)**	**0.37**

Best fitting model is highlighted in bold

**Table 4:** Model fitting results for the paternal negativity Cholesky decomposition

Model	−2LL (DF)	AIC	*χ* ^2^ (df)	*p*
1. Saturated	26316.88 (10371)	5574.88		
2. Cholesky with scalars on all variables	26907.67 (10740)	5422.67		
**2a. Cholesky with scalars on depression and physical aggression**	**26907.68 (10744)**	**5419.68**	**5.01 (4)**	**0.29**

Best fitting model is highlighted in bold

**Table 5:** Model fitting results for the negative life events Cholesky decomposition

Model	−2LL (DF)	AIC	*χ* ^2^ (df)	*p*
1. Saturated	26563.08 (10602)	5359.08		
2. Cholesky with scalars on all variables	27148.15 (10971)	5206.15		
**2a. Cholesky with scalars on depression and physical aggression**	**27153.43 (10975)**	**5203.43**	**5.28 (4)**	**0.26**

Best fitting model is highlighted in bold

#### Maternal negativity

**Fig. 2:**
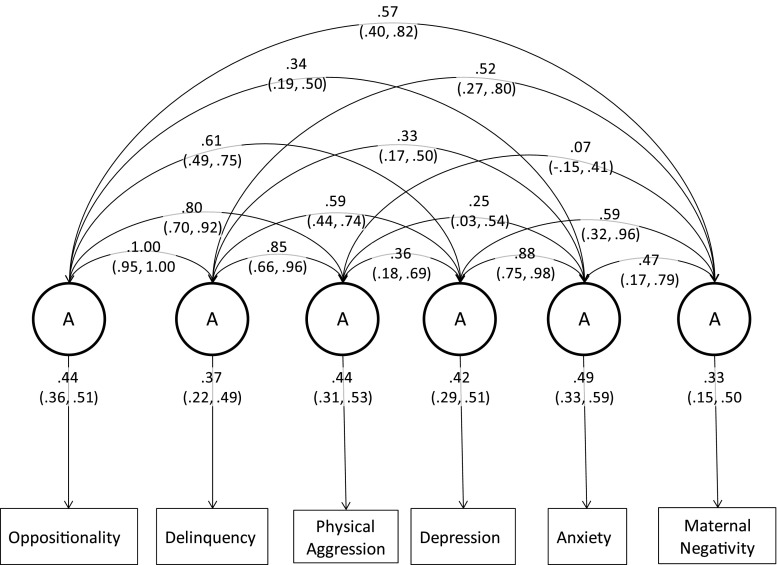
Correlated factors solution showing genetic loadings and genetic correlations for the maternal negativity model (95 % confidence intervals)

The final maternal negativity model included scalars to account for variance differences between the sexes for physical aggression and depression. The genetic components of this model are presented in Fig. 2. The full correlated factors model is included as a table in the Appendix (Table 6). As shown in Fig. 2 maternal negativity had a genetic correlation with oppositionality of 0.57 (95 % confidence interval; 0.40, 0.82), with delinquency of 0.52 (0.27, 0.80), with physical aggression of 0.07 (−0.15, 0.41), with depression of 0.59 (0.32, 0.96), and with anxiety of 0.47 (0.17, 0.79). In the Cholesky decomposition the vast majority of the genetic variance in maternal negativity was accounted for by its association with the behavioural phenotypes included in the model—the residual genetic estimate suggested only 1 % was unaccounted for and the 95 % confidence interval ranged from 0.00 to 0.24, indicating non-significance.

**Table 6:** Maternal negativity: correlated factors solution showing the aetiological overlap between maternal negativity and 5 behavioural phenotypes

	A						C						E					
	1.	2.	3.	4.	5.	6.	1.	2.	3.	4.	5.	6.	1.	2.	3.	4.	5.	6.
1. Oppositionality	0.44 (0.36, 0.51)						0.00 (0.00, 0.06)						0.56 (0.49, 0.63)					
2. Delinquency	1.00 (0.95, 1.00)	0.37 (0.22, 0.49)					−0.59 (−0.77, 0.02)	0.18 (0.09, 0.31)					0.37 (0.29, 0.44)	0.44 (0.38, 0.51)				
3. Physical aggression^a^	0.80 (0.70, 0.92)	0.85 (0.66, 0.96)	0.44 (0.31, 0.53)				−0.27 (−0.64, 0.64)	0.26 (−0.50, 0.88)	0.05 (0.00, 0.15)				0.30 (0.21, 0.38)	0.29 (0.20, 0.39)	0.51 (0.44, 0.59)			
4. Depression^a^	0.61 (0.49, 0.75)	0.59 (0.44, 0.74)	0.36 (0.18, 0.69)	0.42 (0.29, 0.51)			0.02 (−0.43, 0.78	0.56 (0.11, 0.96)	0.76 (−0.53, 1.00)	0.05 (0.01, 0.16)			0.34 (0.26, 0.42)	0.16 (0.07, 0.25)	0.09 (−0.01, 0.19)	0.52 (0.46, 0.60)		
5. Anxiety	0.34 (0.19, 0.50)	0.33 (0.17, 0.50)	0.25 (0.03, 0.54)	0.88 (0.75, 0.98)	0.49 (0.33, 0.59)		0.65 (−1.00, 1.00)	−0.11 (−0.82, 0.72)	−0.44 (−0.99, 0.85)	0.02 (−0.92, 0.89)	0.06 (0.00, 0.20)		0.27 (0.18, 0.35)	0.13 (0.03, 0.22)	0.02 (−0.08, 0.12)	0.47 (0.39, 0.54)	0.45 (0.39, 0.52)	
6. Maternal negativity	0.57 (0.40, 0.82)	0.52 (0.27, 0.80)	0.07 (−0.15, 0.41)	0.59 (0.32, 0.96)	0.47 (0.17, 0.79)	0.33 (0.15, 0.50)	0.06 (−0.27, 0.64)	−0.21 (−0.77, 0.24)	0.18 (−0.77, 0.78)	0.02 (−0.93, 0.67)	−0.16 (−1.00, 0.75)	0.14 (0.02, 0.30)	0.29 (0.20, 0.38)	0.25 (0.15, 0.34)	0.23 (0.13, 0.33)	0.12 (0.02, 0.22)	0.09 (−0.02, 0.19)	0.52 (0.45, 0.61)

Standardised factor loadings (95 % confidence intervals) are given on the diagonals and correlations on the off-diagonals

^a^Scalars were included on physical aggression and depression to account for variance differences between the sexes

#### Paternal negativity

**Fig. 3:**
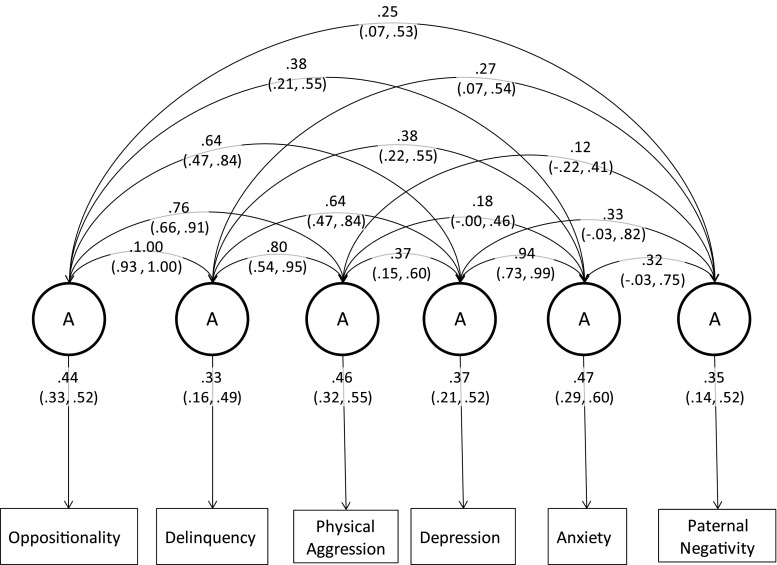
Correlated factors solution showing genetic loadings and genetic correlations for the paternal negativity model (95 % confidence intervals)

The paternal negativity model included scalars on physical aggression and depression. Genetic components of this model are displayed in Fig. 3. The complete model, including shared and non-shared environment estimates are included in Table 7 in the Appendix. As shown in Fig. 3, the genetic correlation between paternal negativity and oppositionality was 0.25 (0.07, 0.53), with delinquency it was 0.27 (0.07, 54), with physical aggression it was 0.12 (−0.22, 0.41), with depression it was 0.33 (−0.03, 0.82), and with anxiety it was 0.32 (−0.03, 0.75). In the Cholesky decomposition a large proportion of the genetic variance in paternal negativity was accounted for by its association with the behavioural phenotypes included: 9 % of variance remained unaccounted for, although the 95 % confidence intervals ranged from 0.00 to 0.44.

**Table 7:** Paternal negativity: correlated factors solution showing the aetiological overlap between paternal negativity and 5 behavioural phenotypes

	A						C						E					
	1.	2.	3.	4.	5.	6.	1.	2.	3.	4.	5.	6.	1.	2.	3.	4.	5.	6.
1. Oppositionality	0.44 (0.33, 0.52)						0.00 (0.00, 0.08)						0.55 (0.48, 0.63)					
2. Delinquency	1.00 (0.93, 1.00)	0.33 (0.16, 0.49)					0.71 (0.49, 0.87)	0.23 (0.10, 0.37)					0.37 (0.29, 0.45)	0.45 (0.38, 0.52)				
3. Physical aggression^a^	0.76 (0.66, 0.91)	0.80 (0.54, 0.95)	0.46 (0.32, 0.55)				0.56 (−0.97, 0.99)	0.59 (−0.75, 1.00)	0.03 (0.00, 0.13)				0.31 (0.22, 0.39)	0.31 (0.21, 0.42)	0.51 (0.43, 0.59)			
4. Depression^a^	0.64 (0.47, 0.84)	0.64 (0.47, 0.84)	0.37 (0.15, 0.60)	0.37 (0.21, 0.52)			0.27 (0.18, 0.93)	0.46 (0.05, 1.00)	0.79 (−0.60, 1.00)	0.10 (0.01, 0.24)			0.34 (0.25, 0.42)	0.15 (0.06, 0.24)	0.09 (−0.01, 0.19)	0.53 (0.45, 0.61)		
5. Anxiety	0.38 (0.21, 0.55)	0.38 (0.22, 0.55)	0.18 (−0.00, 0.46)	0.94 (0.73, 0.99)	0.47 (0.29, 0.60)		−0.24 (−0.80, 1.00)	−0.09 (−1.00, 1.00)	0.06 (−1.00, 1.00)	0.20 (−1.00, 1.00)	0.08 (0.00, 0.24)		0.26 (0.16, 0.35)	0.12 (0.02, 0.21)	0.03 (−0.07, 0.13)	0.45 (0.37, 0.53)	0.45 (0.39, 0.52)	
6. Paternal negativity	0.25 (0.07, 0.53)	0.27 (0.07, 0.54)	0.12 (−0.22, 0.41)	0.33 (−0.03, 0.82)	0.32 (−0.03, 0.75)	0.35 (0.14, 0.52)	−0.17 (−0.51, 0.38)	−0.08 (−0.40, 0.19)	−0.29 (−0.98, 0.58)	0.17 (−0.68, 0.78)	−0.03 (−1.00, 1.00)	0.20 (0.06, 0.36)	0.29 (0.19, 0.39)	0.20 (0.10, 0.30)	0.18 (0.06, 0.29)	0.14 (0.02, 0.25)	0.15 (0.04, 0.26)	0.46 (0.38, 0.54)

Standardised factor loadings (95 % confidence intervals) are given on the diagonals and correlations on the off-diagonals

^a^Scalars were included on physical aggression and depression to account for variance differences between the sexes

#### Negative life events

**Fig. 4:**
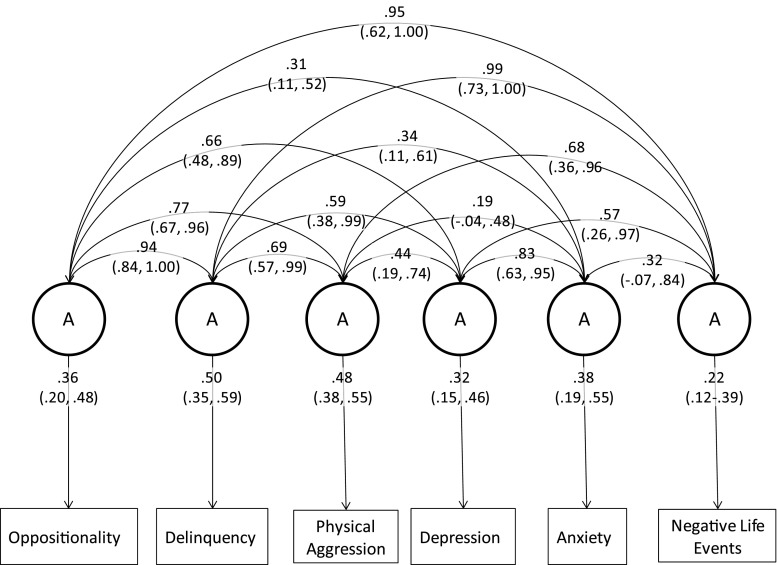
Correlated factors solution showing genetic loadings and genetic correlations for the negative life events model (95 % confidence intervals)

The negative life events model is presented in Fig. 4 and Table 8 in the appendix. Depression and physical aggression included scalars to account for variance differences between the sexes. All genetic variance in negative life events was accounted for by its association with the other phenotypes in the model. The residual genetic variance estimate in the Cholesky decomposition was 0.00 (95 % confidence intervals: 0.00, 0.13). The genetic correlation between negative life events and oppositionality was 0.94 (0.83, 1.00), with delinquency it was 0.99 (0.73, 1.00), with physical aggression it was 0.68 (0.36, 0.96), with depression it was 0.57 (0.26, 0.97), and with anxiety it was.32 (−0.07, 0.83).

**Table 8:** Negative life events: correlated factors solution showing the aetiological overlap between negative life events and 5 behavioural phenotypes

	A						C						E					
	1.	2.	3.	4.	5.	6.	1.	2.	3.	4.	5.	6.	1.	2.	3.	4.	5.	6.
1. Oppositionality	0.36 (0.20, 0.48)						0.06 (0.00, 0.18)						0.58 (0.50, 0.66)					
2. Delinquency	0.94 (0.84, 1.00)	0.50 (0.35, 0.59)					−0.05 (−0.80, 0.71)	0.09 (0.03, 0.21)					0.39 (0.30, 0.47)	0.41 (0.35, 0.48)				
3. Physical aggression^a^	0.77 (0.67, 0.96)	0.69 (0.57, 0.99)	0.48 (0.38, 0.55)				0.52 (−1.00, 1.00)	0.62 (−1.00, 1.00)	0.02 (0.00, 0.07)				0.33 (0.24, 0.41)	0.32 (23, 0.41)	0.51 (0.43, 0.59)			
4. Depression^a^	0.66 (0.48, 0.89)	0.59 (0.38, 0.99)	0.44 (0.19, 0.74)	0.32 (0.15, 0.46)			0.35 (−0.50, 0.76)	0.46 (−0.43, 0.96)	0.32 (−0.60, 1.00)	0.14 (0.04, 0.28)			0.34 (0.25, 0.41)	0.17 (0.06, 0.27)	0.10 (−0.01, 0.20)	0.54 (0.47, 0.62)		
5. Anxiety	0.31 (0.11, 0.52)	0.34 (0.11, 0.61)	0.19 (−0.04, 0.48)	0.83 (0.63, 0.95)	0.38 (0.19, 55)		0.48 (−0.02, 0.89)	−0.10 (−0.90, 0.59)	−0.07 (−0.81, 0.75)	0.66 (0.03, 0.90)	0.16 (0.02, 0.32)		0.25 (0.16, 0.34)	0.11 (0.01, 0.22)	0.04 (−0.07, 0.14)	0.49 (0.41, 0.56)	0.46 (0.40, 0.54)	
6. Negative life events	0.95 (0.62, 1.00)	0.99 (0.73, 1.00)	0.68 (0.36, 0.96)	0.57 (0.26–0.97)	0.32 (−0.07, 0.84)	0.22 (0.12–0.39)	−0.09 (−0.68, 0.34)	0.48 (−0.04, 0.99)	−0.07 (−0.85, 0.82)	0.85 (0.02, 0.98)	0.51 (−0.70, 0.86)	0.20 (0.04, 0.28)	0.18 (0.10, 0.27)	0.20 (0.10, 0.29)	0.16 (0.06, 0.26)	0.15 (0.06, 0.24)	0.13 (0.02, 0.22)	0.59 (0.51, 0.66)

Standardised factor loadings (95 % confidence intervals) are given on the diagonals and correlations on the off-diagonals

^a^Scalars were included on physical aggression and depression to account for variance differences between the sexes

## Discussion

In the present study we set out to investigate the heritability of maternal negativity, paternal negativity and negative life events in an adolescent twin sample. As has been reported previously in this sample (Button et al. 2008) and others (Kendler et al. 1993; Plomin et al. 1990; Saudino et al. 1997), all 3 measures are heritable. Multivariate genetic analyses revealed that this heritability could be accounted for by the association of these environmental stressors with common behavioural and emotional difficulties experienced during adolescence.

The likely generalisability of our results is indicated by our replication of several well established findings. For example, depression and anxiety were both more common in girls than boys, a finding that has been reported several times previously (e.g. Costello et al. 2005; Hankin et al. 1998). As has been found elsewhere (e.g. Tremblay 2010) delinquency was found to increase with age, whereas physical aggression and oppositionality decreased. Correlations between behavioural phenotypes were as would be expected, with higher correlations within the externalising (oppositionality, delinquency, physical aggression) and internalising (depression and anxiety) categories than between them.

Whilst the present study has many strengths (e.g. the inclusion of multiple phenotypes and environmental measures; replication of previous work), we note that our reliance on self-report measures is a limitation. Specifically, shared method variance and perceptual bias may have inflated the phenotypic correlations between measures and using other/multiple informants may have resulted in different patterns of findings. It is unclear how this may have affected the heritability estimates and pattern of genetic correlations between our variables. However, if issues of reporter bias, shared method variance and perceptual bias affect MZ and DZ twins in similar ways then it seems unlikely that they would affect heritability estimates or estimates of genetic correlation between variables.

### Explaining the heritability of environmental measures

We hypothesised that the heritability of parental negativity and negative life events would be explained via their association with oppositionality, delinquency, physical aggression, depression and anxiety. All of these behavioural phenotypes were positively associated with the environmental measures, consistent with the hypothesis that adolescents with emotional and behavioural difficulties tend to be subject to and/or provoke elevated levels of parental negativity and experience an increased number of negative life events.

For maternal negativity, oppositionality, delinquency, depression and anxiety were the phenotypes with which genetic correlations were significant. That is, genetic factors influencing these behaviours were also involved in the experience of maternal negativity. These gene–environment correlations could be active in nature, whereby the adolescent’s genetically influenced behaviour leads them to seek out conflict with their parents, they could be evocative in nature, whereby the adolescent’s genetically influenced behaviour provokes negativity and/or they could be passive, whereby genetic factors that result in negativity on the part of the mother are shared with the adolescent, in whom they lead to oppositional, delinquent, depressive and anxious behaviour. In our Cholesky decomposition no residual genetic variance remained in maternal negativity once that shared with the behavioural phenotypes was accounted for.

For paternal negativity the Cholesky decomposition indicated that 9 % of genetic variance remained after accounting for that shared with the 5 behavioural phenotypes. Confidence intervals included zero, indicating that this was not significant; however this was true for many of the estimates. This could reflect low power and/or may be a result of the small phenotypic correlations between variables. Paternal negativity had significant genetic correlations with oppositionality and delinquency, indicating that these 2 variables were of most importance in explaining the heritability of paternal negativity.

The heritability of negative life events was entirely accounted for in our Cholesky decomposition and genetic correlations in the correlated factors solution were significant for oppositionality, delinquency, physical aggression and depression. This would indicate that genetic factors involved in each of these behavioural phenotypes are also involved in the experience of negative life events. It is noteworthy also that genetic factors involved in the experience of negative life events were almost perfectly correlated with those involved in oppositionality and delinquency.

Oppositionality and delinquency seemed to be the most important behavioural phenotypes in explaining the heritability of all our environmental variables—displaying genetic correlations with each (although it is noteworthy that the genetic correlation between oppositionality and delinquency was almost perfect in every model). Depression was related to maternal negativity and negative life events, with anxiety and physical aggression being genetically related to one environmental variable each. It is intriguing that physical aggression, being a serious problem behaviour, was only weakly related to maternal/paternal negativity and not genetically correlated at all. This could be attributed to the artefactual effects of using a sample displaying only low levels of physical aggression. However aggression was genetically correlated with negative life events, thus physical aggression may in fact not be genetically related to parental negativity at all.

In the present article we have shown that genetic factors play a role in explaining why some adolescents report experiencing environments high in stressors while some report experiencing environments low in stressors. Further, we have shown that this heritability can be explained via the association between these environmental measures and behavioural and emotional problems. There are several ways in which we can interpret this finding. The first explanation is derived from the concepts of active and evocative rGE: whereby genetic factors operate in such a way as to make a person create or seek out an environment that ‘matches’ their genotype. In this interpretation our models would show that genetic factors involved in oppositionality, delinquency, physical aggression, and depression lead a person to increase their likelihood of experiencing negative life events, perhaps through excessive risk taking or an interpersonal style characterised by instability and conflict. This interpretation would also mean that genes involved in oppositionality, delinquency, depression and anxiety in children evoke negativity in their mother. Interestingly this would mean that only genes involved in oppositionality and delinquency evoke negativity in their father, perhaps indicating greater responsiveness to child internalising in mothers than fathers.

The notion that an individual would be genetically predisposed to create for themselves an environment comprising multiple ‘stressors’ may at first glance seem counterintuitive. How could such genes evolve? To take antisocial behaviour as an example, we know that antisocial behaviour is under genetic influence. An individual predisposed to such behaviour will be more successful in some environments than others. Most probably, they will achieve the greatest success in settings in which rule-breaking or aggressive behaviour is (A) accepted (i.e. is unlikely to lead to rejection or other negative consequences) and is (B) associated with positive outcomes. Research has shown that in deviant peer environments antisocial behaviour is associated with popularity (Allen et al. 2005) and dominance (Hawley et al. 2008), and so can be considered to be accepted and associated with positive outcomes in such environments. In this manner something that we may initially conceptualise as a ‘negative life event’—expulsion from school for example—may in fact result in the adolescent leaving an environment that is not matched with their genotype, and in which they are unlikely to achieve success (school) and entering an environment that does match their genotype (a deviant peer environment comprised of other expelled students for example).

An alternative explanation to that of active/evocative rGE is based on the notion of passive rGE. In this scenario genetic factors do not operate directly on the environment but are associated with them indirectly—for example, where individuals with a particular genotype are more likely than others to inhabit a given environment. An example from the current study would be where adolescents genetically predisposed towards depression are more likely to have parents that are prone to negativity because genes involved in parental negativity in parents are involved in adolescent depression in children. In this case the phenotypic association is the spurious result of a shared aetiology.

A noteworthy point here is that whilst a heritable environmental variable is indicative of rGE, it has been argued that for parenting measures in child-based twin samples (such as G1219) passive rGE is actually more likely to load onto C than A (Neiderhiser et al. 2004; Rijsdijk and Sham 2002). This is because in passive rGE the genotype of the parent influences the way that the parent treats their child, independent of the characteristics of the child. As such this is likely to result in parenting that is similar across siblings and does not vary by the genetic relatedness of the siblings (i.e. MZs vs. DZs). We would add that this logic perhaps applies to parent-report parenting in child twin samples more than it does to child-report parenting, as child-report parenting will also be affected by children’s genes. Regardless, it may be more likely that in child twin samples such as ours heritable parenting measures are more indicative of non-passive rGE than passive rGE. That said, in the current study it was not possible to definitively distinguish between types of rGE so we do not advocate one explanation over the other. Indeed, they are not mutually exclusive of one another so it is quite possible that they are each operating in tandem. In order to make distinctions between passive and non-passive forms of rGE it is necessary to combine parent-based twin datasets with child-based twin datasets. Such designs (e.g. the extended-children-of-twins-method; Narusyte et al. 2008; and the technique of comparing twin parent and twin children samples; Neiderhiser et al. 2004) demonstrate that both kinds of rGE may be important.

Parental negativity is associated with a variety of negative outcomes (Rutter et al. 1998), and negative life events are associated with various emotional and behavioural problems (Rowe et al. 2006). As such understanding the role of genetic factors in exposure to such putative environmental risk factors is an important task for researchers. In the present study we have shown that only by considering the accumulative effects of multiple problem behaviours is it possible to understand the role of genetic factors in exposure to environmental stressors. We hope that the present study will assist future researchers in elucidating possible pathways from gene to environment.

## Appendix

See Tables 3, 4, 5, 6, 7, 8
